# Quercetin Inhibits Hephaestin Expression and Iron Transport in Intestinal Cells: Possible Role of PI3K Pathway

**DOI:** 10.3390/nu15051205

**Published:** 2023-02-28

**Authors:** Hanuma Naik Ramavath, Venu Konda, Raghu Pullakhandam

**Affiliations:** Drug Safety Division, ICMR-National Institute of Nutrition, Jamai Osmania, Hyderabad 500007, Telangana, India

**Keywords:** polyphenols, quercetin, Caco-2 cells, iron transporters, hephaestin, ferroportin, iron, zinc, PI3K

## Abstract

Previous studies demonstrated that quercetin, a polyphenolic compound, inhibits the transport of iron by down-regulation of ferroportin (FPN1), an iron export protein. We have previously demonstrated that activation of the PI3K signaling pathway by zinc stimulates the intestinal iron uptake and transport by stimulating the expression of iron regulatory protein 2 (IRP2) dependent divalent metal iron transporter 1 (DMT1, apical iron transporter) expression and caudal-related homeobox transcription factor 2 (CDX2) dependent hephaestin (HEPH, basolateral ferroxidase required for iron oxidation) expression, respectively. Since polyphenols are antagonists of the PI3K pathway, we hypothesized that quercetin might inhibit basolateral iron transport via the down-regulation of hephaestin (HEPH). Here in we investigated the effect of quercetin on iron uptake, transport, and expression of iron transporters in intestinal cells. In differentiated Caco-2 cells grown on permeable supports, quercetin inhibited the basolateral iron transport while increasing the iron uptake, possibly due to higher cellular retention. Further, quercetin down-regulated the protein and mRNA expression of HEPH and FPN1 but not that of IRP2 or DMT1. In addition, quercetin also abrogated the zinc-induced Akt, CDX2 phosphorylation, and HEPH expression. Together these results suggest that inhibition of iron transport by quercetin is mediated via the down-regulation of CDX2-dependent HEPH expression via inhibition of the PI3K pathway.

## 1. Introduction

Iron is a micronutrient required for growth and health. Systemic iron homeostasis is achieved by regulating intestinal iron absorption [1,2,3]. There are two types of dietary iron; heme and non-heme iron, the latter being predominant in typical vegetarian diets [4,5]. At the apical membrane of enterocytes, divalent metal ion transporter 1 (DMT1) facilitates the uptake of ferrous iron [4]. In the enterocytes, iron is either stored in ferritin or transported to the serosal side via hephaestin (HEPH)/ferroportin (FPN1) mediated export [3,6]. The duodenal cytochrome-B (DcytB) reduces the ferric iron at the apical surface, while HEPH oxidizes the ferrous iron at the basolateral side of enterocytes [4,6]. Depending on the iron status of intestinal cells, the iron regulatory proteins 1 and 2 (IRP1 and IRP2) modulate the mRNA translation of iron transport and storage proteins [2]. Alternatively, hepcidin, an iron hormone elevated during high iron stores, inhibits the iron absorption from enterocytes and egress from the liver and macrophages by inducing internalization and degradation of FPN1 [1,7].

Apart from the iron status of the host, numerous dietary factors modulate intestinal iron absorption. For instance, ascorbic acid (vitamin C) stimulates non-heme iron absorption, while phytic acid and polyphenols inhibit the same [8]. Clinical studies reported that iron absorption is reduced when it is consumed along with polyphenol-rich foods [9,10,11]. Chelation of dietary iron by anionic polyphenols is thought to be the potential mechanism [12]. However, studies in intestinal cells showed that grape seed polyphenols specifically inhibit the transport of both non-heme and heme-iron [13,14]. Similarly, quercetin has been shown to reduce iron transport via down-regulation of FPN1 expression via a miRNA-dependent mechanism [15,16]. Further, intraperitoneal administration of quercetin stimulated the liver hepcidin mRNA expression in rats [16]. We have previously shown that the PI3K pathway is involved in the regulation of uptake and basolateral transport of iron in intestinal cells [17,18,19]. Activation of the PI3K pathway by zinc stimulated the iron uptake and transport by IRP2-dependent induction of DMT1- and CDX2-dependent induction of HEPH expression, respectively [17]. Interestingly, polyphenols, including quercetin, are inhibitors of the PI3K pathway [20,21]. Therefore, we hypothesized that quercetin-induced inhibition of iron transport in intestinal cells could involve modulation of PI3K-dependent HEPH expression.

The purpose of the current study was to investigate the effect of quercetin on intestinal iron transport, particularly the intermediary role of HEPH, if any, in differentiated Caco-2 cells, a model for intestinal cells. 

## 2. Materials and Methods

Materials: The culture media, antibiotic-mycotic mix, and trypsin were obtained from *invitrogen* (Carlsbad, CA, USA). HEPH and DMT1 antibodies were from Santacruz biotechnology (Dallas, TX, USA). pAkt (ser 473), CDX2 antibodies were from Cell Signalling Technology (Danvers, MA, USA). FPN1 antibody was from Alpha Diagnostics (Cambridge, MA, USA). ^55^Fe was obtained from American Radio Chemicals, (Saint Louis, MO, USA). All other reagents, including β –actin antibody and minimum essential medium (MEM) containing L-glutamine, were procured from Sigma Chemical Co. (Bangalore, India) unless specified.

### Methods

Caco-2 cell culture: The Caco-2 cell line (HTB-37) was obtained from the American Type Culture Collection (ATTC, and Rockville, MD, USA) and grown as described previously [17,19]. Briefly, cells were grown in 6 well plates or transwell plates, in MEM containing 10% (*v*/*v*) fetal bovine serum (FBS, heat-inactivated) and 1% (*v*/*v*) antibiotic-mycotic mixture. The cells were grown in an incubator (Thermo, Waltham, MA, USA) at 37 °C temperature, 5% CO_2_ and 95% humidity. The medium was changed every alternate day. Cells were subcultured at 70–80% confluence. The cells were used for the experiments 21 days post-seeding to ensure spontaneous differentiation.

Treatments and cell lysis: A day before the treatments, the cells were washed with 10 mmol/L phosphate buffer saline pH 7.2 (PBS), and the spent media was replaced with serum-free MEM. Next day cells were treated with quercetin (100 µmol/L) and/or Zn (100 µmol/L) for indicated times (0 to 24 h). Wherever present quercetin was added 2 h prior to the zinc treatment. After the treatments, the cells were rinsed thrice with ice-cold PBS and lysed in Cell Lytic M containing protease (1X) and phosphatase (1X) inhibitor cocktail. The micro-BCA kit was used to estimate the protein concentration in cell lysates. 

^55^Fe uptake and transport: For the transport studies, the Caco-2 cells were grown on 6—well-transwell inserts (0.3 μm pore size, Corning, New YORK, NY, USA) and used after 21 days of seeding as described previously [18,22]. Briefly, after the treatment with quercetin (24 h), the cells were washed with buffer (10 mmol/L HEPES, pH 6.5 containing NaCl 140 mmol/L; KCl 5 mmol/L; Na_2_HPO_4_ 1 mmol/L; CaCl_2_ 1 mmol/L; MgCl_2_ 0.5 mmol/L; D-glucose 5 mmol/L) and incubated in the same buffer for 10 min, while the basal chamber contained 2.5 mL of MEM with 0.5% FBS. To assess the uptake and transport, the apical chamber buffer was supplemented with 10 µmol/L FeCl_3_ (traced with 1.1 µcurie/mL ^55^FeCl_3_) and 1 mmol/L ascorbic acid (prepared fresh in 0.1N HCl). At the end of 1 h, ^55^Fe radioactivity in basolateral media and in cells was estimated by β-scintillation counter (Perkin Elmer, Downers Grove, IL, USA) as described previously [22].

Immunoblotting: Equal concentration of protein (20–30 mg) was denatured in sample buffer and subjected to electrophoresis on SDS-gels under reducing conditions. The gels were transblotted onto the nitrocellulose membranes, blocked (5% non-fat dry milk or BSA for 1 h), and incubated with primary and secondary antibodies, respectively. The blots were developed with chemiluminescence detection kit (Bio-Rad, Hercules, CA, USA) and images were captured on G-box imaging system (Frederick, MD, USA). The same blots were stripped and re-probed with β -actin, served as a loading control. The densities were quantified using image-J software (NIH, Bethesda, MD, USA) and normalized to respective loading control densities.

Real time PCR: Following the treatment, the total RNA was isolated from cells using TRIzol, and treated with DNase. After cDNA synthesis, mRNA levels of HEPH, FPN1 and β-2 microglobulin (used as a housekeeping gene) were analyzed by real-time PCR using power SYBR Green PCR kit (Bio-Rad, Hercules, CA, USA) on a Bio-Rad CFX thermocycler. The relative gene expression was computed using ∆Ct method. The primer sequences were reported elsewhere [18,19]. The normalized data (housekeeping gene) are presented as the mean ± S.E.M.

Statistics: Statistical analysis was carried out using Sigma Plot (version 10.0). Unpaired t-tests or One-way ANOVA followed by Tukey’s post hoc test was used to compare the differences between groups, and *p* < 0.05 was considered significant.

## 3. Results 

**Figure 1:**
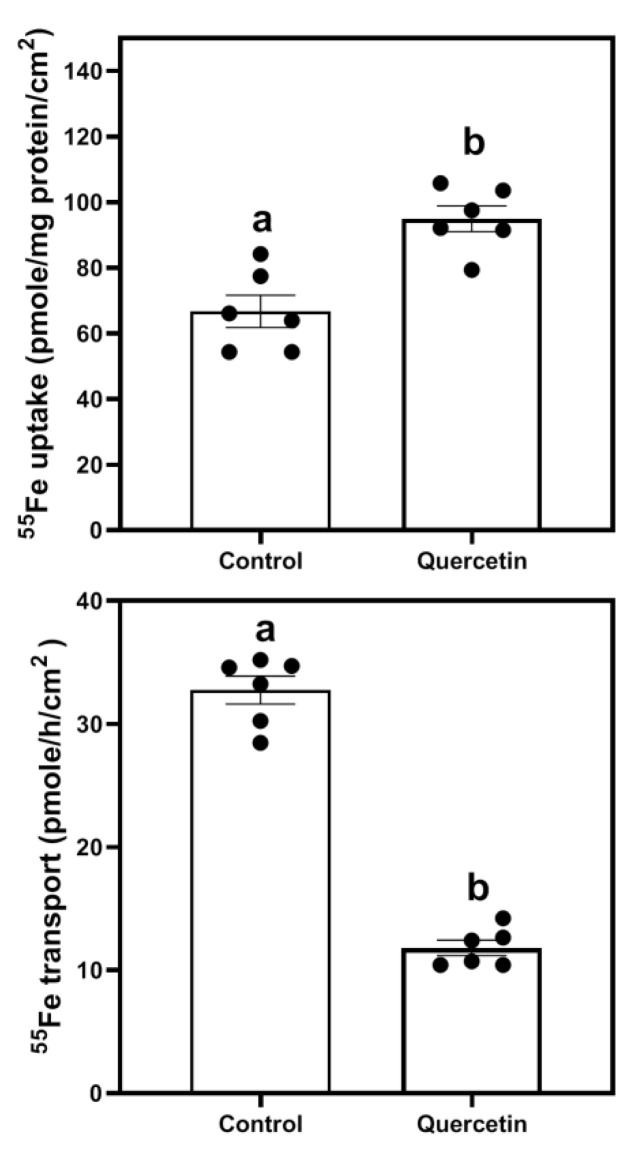
Effect of quercetin on ^55^Fe iron uptake and transport in Caco-2 cells: Differentiated Caco-2 cells grown in transwell plates were incubated with quercetin (100 µmol/L) for 24 h, after which iron uptake (**top panel**) and transport (**bottom panel**) were measured as described in methods. The experiments were performed in triplicate and repeated twice to generated 6 independent observations (*n* = 6). The bars (mean ± SEM) without common superscripts differ significantly (*p* < 0.05), unpaired *t*-test.

In order to determine whether quercetin was modulating iron absorption in intestinal cells, the effect of quercetin on ^55^Fe uptake and transport in differentiated Caco-2 cells grown in transwell plates was measured. Quercetin treatment significantly increased the iron uptake (cell-associated ^55^Fe activity, Figure 1, top panel), while reducing the basolateral efflux (^55^Fe appeared in basolateral media, Figure 1, bottom panel) compared to control cells. Moreover, the ratio of cell associated ^55^Fe with that of basolateral media was 4-fold higher in quercetin-treated cells compared to untreated control cells.

**Figure 2:**
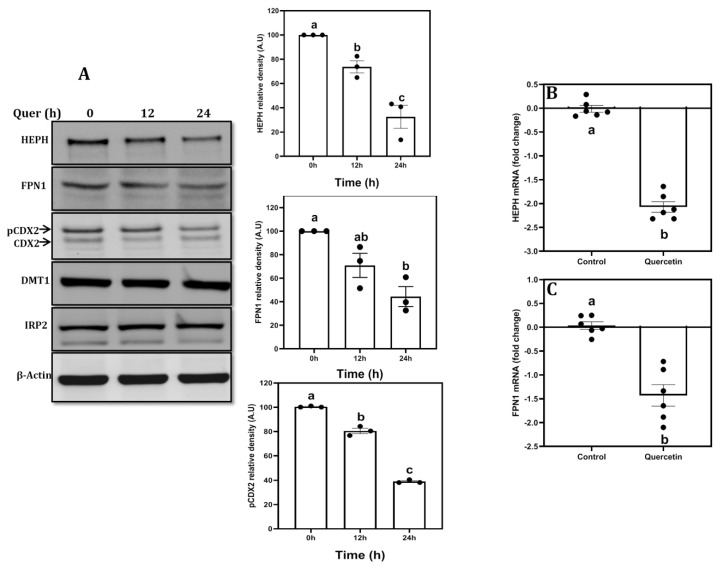
Effect of quercetin on expression of iron transporters in Caco-2 cells: Differentiated Caco-2 cells were treated with quercetin (100 µmol/L for 0, 12 and 24 h), after which the protein (**A**) and mRNA (24 h), (**B**,**C**) expression of HEPH (~130 kDa), FPN1 (~62.5 KDa), CDX2 (~34 KDa), DMT1 (~62 KDa), IRP2 (90 kDa) were measured by western blotting or qPCR. The densities were normalized to the β-actin, loading control. The bars (mean ± SEM) without common superscripts differ significantly (*p* < 0.005); one way ANOVA, post hoc Tukey’s test.

To further understand the mechanism, we measured protein (DMT1, HEPH, FPN1, IRP2 and CDX2) and mRNA (DMT1, HEPH and FPN1) expression of iron transporters. Quercetin treatment significantly reduced HEPH and FPN1 but not that DMT1 or IRP2 protein expression as a function of time at 12 and 24 h (Figure 2A). In agreement, quercetin also significantly inhibited the HEPH and FPN1 mRNA expression compared to untreated controls (Figure 2B,C). However, DMT1 mRNA expression remained unaffected (data not shown). Further, quercetin also significantly inhibited the phospho-CDX2 (pCDX2, slower migrating upper band), while the unphosphorylated CDX2 (bottom band) levels remained similar compared to untreated controls (Figure 2A). 

**Figure 3:**
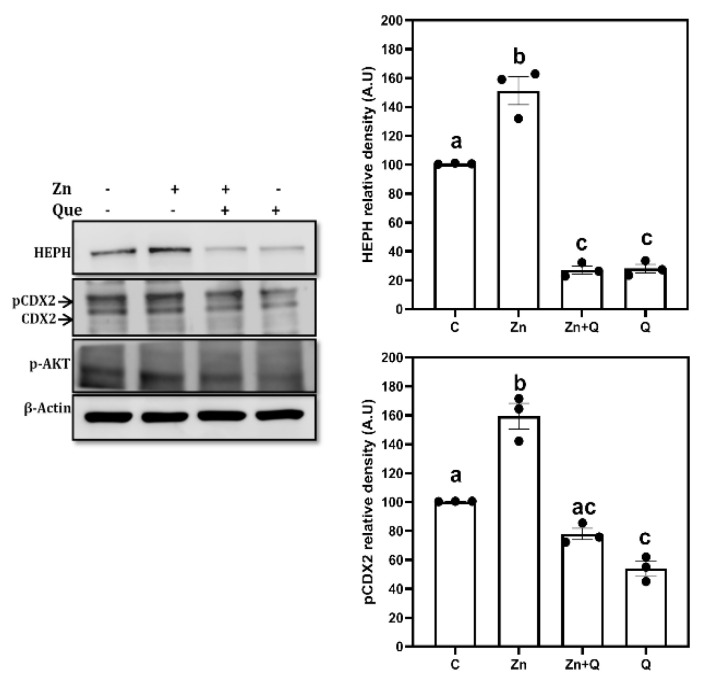
Effect of Zinc and/or Quercetin on expression of hephaestin and CDX2 in Caco-2 cells: Differentiated Caco-2 cells were treated with quercetin (Q, 100 µmol/L) and/or Zn (100 µmole/L) for 12 h, after which the protein expression of HEPH (~130 kDa), CDX2 (~34 kDa) and pAkt (p-Ser 473, ~60 kDa) were measured by western blotting. Wherever present quercetin was added 2 h prior to the Zn. The densities were normalized to the β-actin, loading control. The bars (mean ± SEM) without common superscripts differ significantly (*p* < 0.05); one way ANOVA, post hoc Tukey’s test.

We have previously reported that Zn induced iron transport and HEPH expression in enterocytes is regulated by PI3K dependent CDX2 phosphorylation [18,19]. Therefore, we tested the effect of quercetin on zinc induced CDX2 phosphorylation and HEPH expression. Zinc treatment resulted in significant induction of pCDX2 (upper band) and HEPH expression compared to untreated control cells, all of which are significantly reversed in the presence of quercetin (Figure 3). Quercetin also inhibited the zinc induced Akt (p-Ser 473) phosphorylation. Further, both pCDX2 and HEPH expression in quercetin treated cells was significantly lower compared to untreated control cells (Figure 3).

## 4. Discussion

Consumption of polyphenol-rich foods is known to inhibit intestinal iron absorption [9,10]. Chelation of non-heme iron by anionic polyphenols is thought to be the underlying mechanism, but few studies demonstrated that polyphenols such as quercetin could block iron export from intestinal cells to the serosal compartment by inhibiting FPN1 expression via the miRNA-dependent mechanism [13,14,15,23]. However, the effect of quercetin on HEPH remains to be studied. In the current study, we demonstrated that quercetin down-regulates the iron transport and expression of basolateral iron transporters HEPH and FPN1. In addition, we also demonstrated that quercetin inhibits zinc-induced CDX2 phosphorylation and HEPH expression. These results suggest that reduced iron transport due to quercetin also involves down-regulation of HEPH expression (in addition to FPN1), which could be mediated via inhibition of PI3K-dependent CDX2 phosphorylation. 

Previous studies in intestinal cells showed that polyphenols from tea and grape seed extract inhibit the basolateral transport of both non-heme and heme iron [15,24,25]. It was also shown that quercetin down-regulates the expression of FPN1 via the miRNA-dependent mechanism [15]. Since the FPN1 is the only known iron exporter so far known, its down-regulation by quercetin could be mechanistically linked with reduced iron transport in intestinal cells. However, oxidation of intracellular iron by HEPH is required for FPN1-mediated basolateral iron exit from intestinal cells [6]. In a previous study, we showed that induction of HEPH expression by zinc is sufficient to stimulate iron transport even when FPN1 levels remain unchanged [18,19]. However, the effect of quercetin on HEPH expression and underlying mechanisms remains to be explored. Therefore, we tested whether quercetin regulates iron transport and iron transporter expression in differentiated Caco-2 cells. Quercetin increased the iron uptake but reduced the iron transport, consistent with previous studies in intestinal cells and rat models [13,14,15]. In agreement, quercetin also reduced the mRNA and protein expression of both HEPH and FPN1. Interestingly, quercetin had no effect on DMT1 expression; therefore, increased iron uptake due to quercetin could be a reflection of higher retention of absorbed iron due to specific down-regulation of basolateral iron transport rather than increased uptake of iron.

Quercetin did not influence the IRP2 or DMT1 levels but specifically reduced the CDX2 phosphorylation and expression of HEPH levels. A high correlation of CDX2 with HEPH levels has been observed in animal and human tissues, and multiple CDX2 binding sites have been identified in the HEPH gene promoter region [24,25]. Further, ectopic expression of CDX2 induced the HEPH expression, and its silencing reduced the HEPH expression. Moreover, phosphorylation of CDX2 is required for its transcriptional activation as well as proteasomal degradation [26]. We have previously shown that zinc-induced up-regulation of HEPH expression is inhibited by PI3K antagonist LY294002 [19]. Since quercetin is reported to inhibit PI3K activity in different cell types [20,21], inhibition of zinc-induced PI3K activation by quercetin might block the subsequent pCDX2-HEPH pathway. The fact that quercetin also inhibited the zinc-induced Akt phosphorylation, as well as zinc-induced iron transport (Supplementary Figure S1), renders further support to this notion. To the best of our knowledge, this is the first demonstration that quercetin inhibits CDX2 phosphorylation in intestinal cells.

We believe that the above results have important clinical and mechanistic implications. The mechanism by which polyphenols inhibit iron absorption in general or quercetin, in particular, could be at multiple levels, such as chelation and inhibition of absorbed iron export to the serosal side, which in turn could be excreted through exfoliation of intestinal cells into feces. Therefore, clinical studies measuring the acute effects of polyphenols or quercetin might underestimate the long-term consequences of quercetin exposure on iron absorption/status. Since quercetin is very abundant in commonly consumed foods, it warrants further studies aimed at understanding their long-term exposure to iron absorption/status, including the effects on systemic iron homeostasis (i.e., serum hepcidin). In contrast, dietary polyphenol supplementation was reported to improve erythropoiesis in both healthy and subjects with metabolic disease [27]. Therefore, the acute effects of purified quercetin on intestinal iron absorption observed in this study cannot be generalized to whole fruits and vegetables. It is also noteworthy that the vitamin C present in fruits and vegetables reported offsetting the effects of polyphenols on iron absorption [28]. Interestingly, while the activation of the PI3K pathway by zinc stimulated both the uptake and transport of iron in intestinal cells, its inhibition by quercetin appears to specifically inhibit intestinal iron absorption by selectively blocking the basolateral transport. Therefore, mechanistically PI3K pathway can be a potential target for both improving (i.e., anemia) and reducing (i.e., iron overload) iron status. However, considering the potential differences in signaling pathways in cancerous compared to primary cells, these results need to be validated in other intestinal cell models (i.e., HSIEpC, human small intestine epithelial cells).

In summary, the above results suggest that quercetin inhibits iron transport from intestinal cells via down-regulation of HEPH in addition to FPN1 expression in intestinal cells, possibly by inhibiting the PI3K-CDX2 pathway. These findings suggest a possibility of exploiting the PI3K pathway either for improving or reducing iron absorption. 

## Data Availability

The data presented in this study are available upon justified request to the corresponding author.

## Supplementary Materials

The following are available online at https://www.mdpi.com/article/10.3390/nu15051205/s1, Supplementary Figure S1: Effect of quercetin on zinc induced iron transport.
