# Supplementary material for: Quercetin Inhibits Hephaestin Expression and Iron Transport in Intestinal Cells: Possible Role of PI3K Pathway
Source: Nutrients. 2023 Feb 28;15(5):1205. doi: 10.3390/nu15051205 (PMC10005583; doi:10.3390/nu15051205)
Supplement: Supplementary file 1 [file nutrients-15-01205-s001.zip › nutrients-2196496-supplementary.pdf]

**Supplementary Figure**

# Quercetin inhibits hephaestin expression and iron transport in intestinal cells: Possible role of PI3K pathway

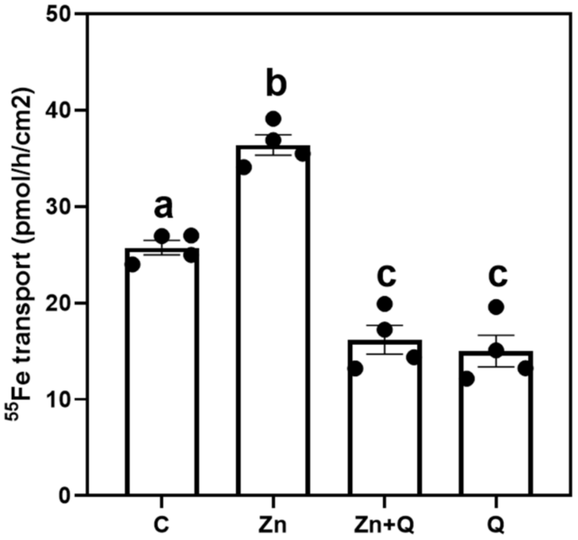


**Supplementary Figure S1. Effect of quercetin on zinc induced iron transport**: Differentiated Caco-2 cells grown in transwell plates were pre-treated with quercetin 100 µmol/L quercetin followed by Zn (100 µmol/L) for 24 h, after which $^{55}Fe$ iron transport was measured as described in methods section. The experiment was performed in quadruplicates. The bars are mean ± SEM and the bars without common superscripts differ significantly ($p < 0.05$); one way ANOVA, post-hoc Tukey's test.
